# SOHPIE: statistical approach via pseudo-value information and estimation for differential network analysis of microbiome data

**DOI:** 10.1093/bioinformatics/btad766

**Published:** 2023-12-22

**Authors:** Seungjun Ahn, Somnath Datta

**Affiliations:** Department of Population Health Science and Policy, Icahn School of Medicine at Mount Sinai, New York, NY 10029, United States; Department of Biostatistics, University of Florida, Gainesville, FL 32610, United States

## Abstract

**Summary:**

The SOHPIE R package implements a novel functionality for “multivariable” differential co-abundance network (DN, hereafter) analyses of microbiome data. It incorporates a regression approach that adjusts for additional covariates for DN analyses. This distinguishes from previous prominent approaches in DN analyses such as MDiNE and NetCoMi which do not feature a covariate adjustment of finding taxa that are differentially connected (DC, hereafter) between individuals with different clinical and phenotypic characteristics.

**Availability and implementation:**

SOHPIE with a vignette is available on CRAN repository https://CRAN.R-project.org/package=SOHPIE and published under General Public License (GPL) version 3 license.

## 1 Introduction

An emerging body of evidence indicate that the microbiome plays a critical and causal role in human health and disease (Mohajeri *et al.* 2018, Ogunrinola *et al.* 2020). Recent advancements in next-generation sequencing platforms have enabled researchers for the comprehensive analysis of microbial diversity (Reuter *et al.* 2015, Durazzi *et al.* 2021), most commonly estimated by an operational taxonomic units. For instance, in the last decade, large-scale studies such as the Human Microbiome Project (Turnbaugh *et al.* 2007) and Metagenomics of the Human Intestinal Tract (Qin *et al.* 2010) have been conducted to unfold the link between human microbiome and health.

Nonetheless, many statistical challenges remain for the analysis of microbiome data, mainly due to compositional (Aitchison 1982) and sparse data structure (Bharti and Grimm 2021). One of the most recent challenges is to unravel the microbial co-abundances among taxa through differential co-abundance network (DN) analysis (Matchado *et al.* 2021). The DN analysis is a method derived from the network theory that compares the topological properties (e.g. centrality or connectivity) between two or more networks (or graphs) under different biological conditions of individuals (e.g. high-risk versus low-risk groups).

To date, two statistical methods have been developed for the DN analysis, namely Microbiome Differential Network Estimation (MDiNE; McGregor *et al.* 2020) and Network Construction and comparison for Microbiome data (NetCoMi; Peschel *et al.* 2021). However, a covariate adjustment is not available in these methods. This factor hinders the possibilities for researchers to explore any additional clinical or demographic information that may be associated with the microbial composition and human host.

In an effort to fill the gap, we have recently proposed the SOHPIE–DNA, a novel pseudo-value regression approach (Ahn and Datta 2023) in analyzing microbiome data, based on a direct marginal modeling with jackknife resampling technique (Efron and Tibshirani 1993, Andersen *et al.* 2003). SOHPIE–DNA allows for a direct inclusion of covariates as independent variables in a regression model and has been shown to improve performance metrics over two existing methods (namely MDiNE and NetCoMi) in simulation studies.

In this article, we feature an R package, named ‘**S**tatistical Appr**O**ac**H** via **P**seudo-value **I**nformation and **E**stimation’ (SOHPIE; pronounced as *Sofie*) for DN analysis of microbiome data. SOHPIE is a software expansion to the methodological framework (SOHPIE–DNA) for wider reproducibility and accessibility. Herewith, we describe an algorithm in Software Implementation section, followed by a brief tutorial with built-in test data from the American Gut Project (McDonald *et al.* 2018) and the Diet Exchange Study (O’Keefe *et al.* 2015) in Section 3.

## 2 Software implementation

The SOHPIE has dependencies to the packages available in the Comprehensive R Archive Network (CRAN) library including robustbase, dplyr, fdrtool, and gtools. In addition, the base R packages such as parallel and stats.

**Figure 1.**
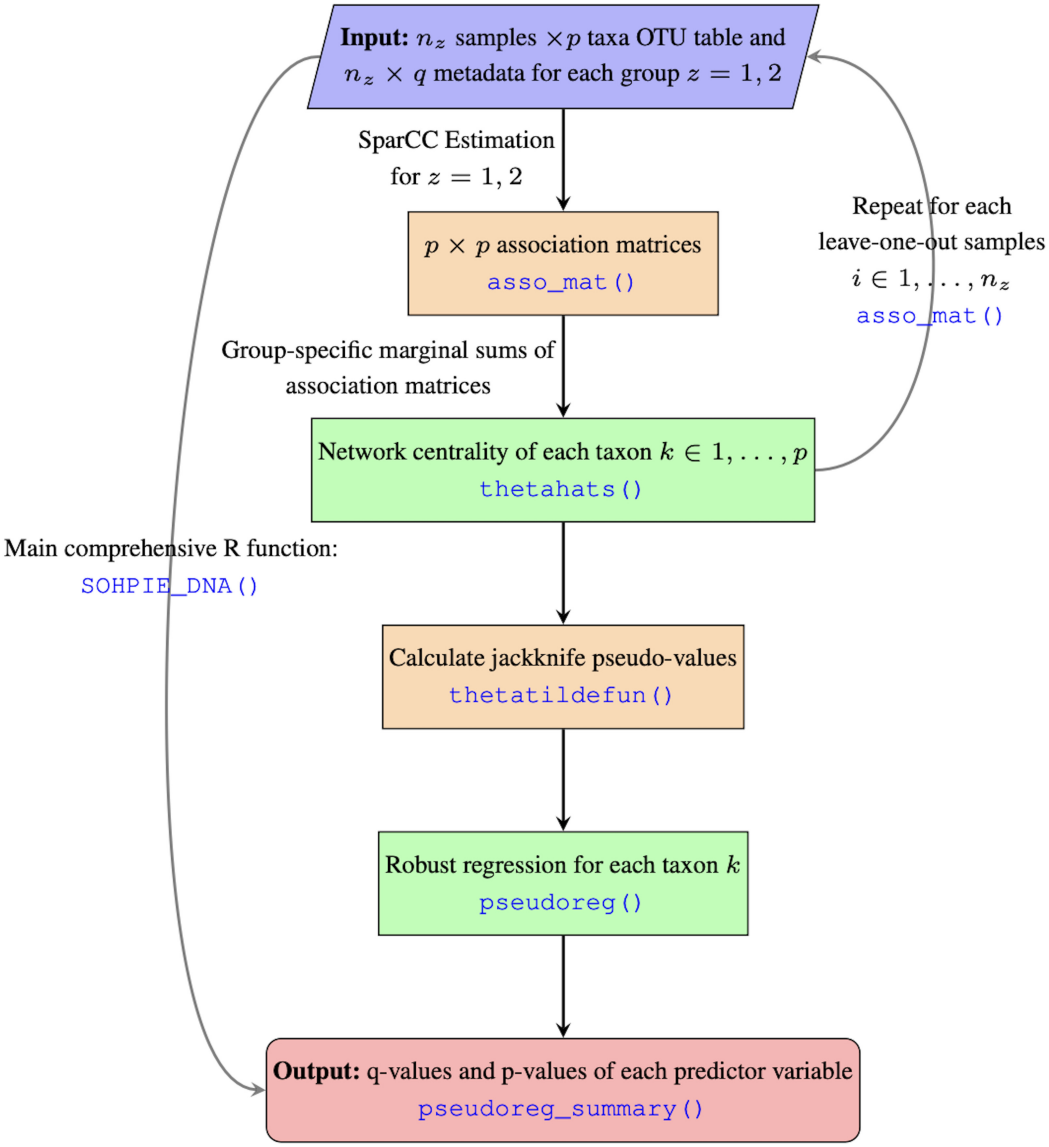
A flow diagram to describe the algorithmic framework to perform a DN analysis with a pseudo-value regression approach in SOHPIE R package. Blue texts indicate the name of R functions specific to each module in SOHPIE. Although a user can execute each separate functions for a pseudo-value regression analysis, it is highly recommended to use one single function, called SOHPIE_DNA(), for convenience.

The package provides a complete framework for the DN analysis in analyzing microbiome data. It comprises of five modules: (i) estimation of co-abundance network (or association matrix, which is a symmetric matrix of pairwise measures of association between two sets of taxa) and calculation of network centrality for the whole data; (ii) repeat module (i) but for each leave-one-out samples; (iii) calculation of jackknife pseudo-values; (iv) robust regression with jackknife pseudo-values as response variable and one or more covariates; and (v) extraction of regression analysis results such as *q*-values (Storey 2002) of each predictor variable. Of note, SparCC (Friedman and Alm 2012) was used for the estimation of co-abundance network. The wrapper function for SparCC was acquired from CCLasso (Fang *et al.* 2015), provided in GitHub (https://github.com/huayingfang/CCLasso). See Figure 1 for illustrative presentation of our work flow.

## 3 Application

We demonstrate the functionalities of SOHPIE using two test data included in the package: combinedamgut and combineddietswap. Each of combinedamgut and combineddietswap are subsets of the pre-processed datasets that are available in SpiecEasi (Kurtz *et al.* 2015) and microbiome (Lahti and Shetty 2017) R packages, respectively.

### 3.1 Example I: American Gut Project Data

In SOHPIE, there is a vignette to assist users in performing the DN analysis with a pseudo-value regression approach. This details a step-by-step description of the process using combinedamgut data. A vignette, provided as Supplementary Data, can be viewed on CRAN page or from the R console by typing browseVignettes(”SOHPIE”).

After the data is loaded, a user should obtain indices for each category of main binary variable, separately (e.g. living with versus without a dog). Then, the main function, SOHPIE_DNA(), is employed for the DN analysis. It requires the name of previously loaded dataset and also the indices for each category that are obtained earlier. Additionally, a user must specify a value of trimming proportion c∈{0.5,1} for the least trimmed squares (LTS) estimator of the robust regression. We have used *c *=* *0.5 throughout this article, a default value suggested in robustbase package. In Supplementary Data, a small sensitivity analysis was conducted to investigate how sensitive the fit to the choice of the trimming proportion (suggested by a referee). Overall, the performances seem to be robust in this example with respect to the choice of *c*. Further information about a choice of trimming proportion and LTS estimator can be found elsewhere (Rousseeuw 1984, Pison *et al.* 2002). SOHPIE_DNA() outputs a list of data.frame objects containing coefficient estimates, *P*-values, and *q*-values of each predictor variable from the pseudo-value regression fitted for each taxon.

For user convenience, there are functions to quickly retrieve the names of differentially connected (DC) taxa (DCtaxa_tab()), *P*-values, *q*-values, coefficient estimates, and standard error of coefficient estimates of all variables (pval(), qval(), coeff(), and stderrs()) or for a specific variable of interest (pval_specific_var(), qval_specific_var(), coeff_specific_var(), and stderrs_specific_var()) that are considered in the analysis. A detailed information of the usage and demonstration can be found in the vignette.

### 3.2 Example II: Diet Exchange Study Data

In this example, we take a slight detour to account for temporal changes of connectivity of taxa in the analysis of combineddietswap dataset. More description is provided in the vignette (see Supplementary Data).

In the original study (O’Keefe *et al.* 2015), the dietary intervention was designed to assess the level of fat and fiber intake among study participants with high versus low colon cancer from two geographically distinct regions: African-Americans from Pittsburgh, Pennsylvania (AAM) versus rural South Africans (AFR), respectively. The study participants had undergone an endoscopy at baseline and at 29 days after the dietary intervention.

As a preparation step, the indices are located for each setting (i.e. AAM and baseline, AFR and baseline, AAM and 29 days, and AFR and 29 days). The analysis begins by estimation and re-estimation of association matrices for each setting using asso_mat(). For each geographic group (AAM and AFR) separately, the difference of estimated (and re-estimated) association matrices between two time points are observed. Then, these matrix differences are used for the calculation of network connectivity (thetahats()) and jackknife pseudo-values (thetatildefun()). The last component of this example is to fit the pseudo-value regression with covariates using pseudoreg(). Further, pseudoreg.summary() is used to produce a list of data.frame objects for coefficient estimates, *P*-values, and *q*-values of each predictor.

## 4 Conclusion

SOHPIE implements a suite of functions facilitating differential network analysis of finding DC taxa between two heterogeneous groups. The key features are the ability to appropriately to test for differential connectivity of a co-abundance network and also to adjust for covariates by introducing a pseudo-value regression framework. The jackknife-generated pseudo response values for regression reflect the influence of the *i*-th sample on the centrality (i.e. connectivity scores) of each taxon. The regression model describes the “effect” of the main factor (binary group variable) *Z* and covariates *X* on the quantified influences. Thus, DC between two groups is described and quantified by the regression coefficient on *Z*, in terms of how much the grouping affect the influences on the centrality, adjusting for other covariates. SOHPIE is a user-friendly and open-source software tool.

## Supplementary Material

btad766_Supplementary_Data

## Data Availability

The SOHPIE R package is freely available in the CRAN: https://CRAN.R-project.org/package=SOHPIE. The two sample datasets (combinedamgut and combineddietswap) are included in SOHPIE. More information on the original studies and data sources are stated in the main text above.
