# Supplementary material for: SOHPIE: statistical approach via pseudo-value information and estimation for differential network analysis of microbiome data
Source: Bioinformatics. 2023 Dec 22;40(1):btad766. doi: 10.1093/bioinformatics/btad766 (PMC10807904; doi:10.1093/bioinformatics/btad766)
Supplement: btad766_Supplementary_Data [file btad766_supplementary_data.zip › SupplementaryFile1_SOHPIE_Vignette.pdf]

An Introduction to SOHPIE

Seungjun Ahn

October 20th, 2023

We introduce a Statistical Approach via Pseudo-value Information and Estimation (SOHPIE; pronounced as "Sofie"). This is a regression modeling method for differential network (DN) analysis that can include covariate information in analyzing microbiome data. SOHPIE is a software extension of our methodology work [1].

Requirements

Please install these R packages prior to use SOHPIE.

```
# library(robustbase) # To fit a robust regression.
# library(parallel) # To use mclapply() when reestimating the association matrix.
# library(dplyr)  # For the convenience of tabulating p-values, coefficients, and q-values.
# library(fdrtool) # For false discovery rate control.
# library(gtools) # To estimate an association matrix via SparCC.
```

```
library(SOHPIE)
```

Load the study data from SOHPIE R package:

Two sample datasets are available in this package. One (`combinedamgut`) is from the American Gut Project [2] and contains 138 taxa and 268 subjects. In this vignette, the first 30 out of 138 taxa will be used for the simple demonstration purpose. The other (`combineddietswap`) is from the geographical epidemiology study of diet swap intervention [3] that includes 112 taxa with 37 subjects (20 African Americans from Pittsburgh and 17 rural South Africans). The full data of each study are available in the SpiecEasi and microbiome R packages, respectively.

Example I: American Gut Project Data

```
set.seed(20050505)
data(combinedamgut) # A complete data containing columns with taxa and clinical covariates.
```

Data processing for the toy example using sample dataset from American Gut Project:

The main grouping variable will be the indicator variable for the status of living with a dog. After the data processing, the indices of subjects will be available for each 'Not living with a dog (Group A)' vs. 'Living with a dog (Group B).' We need these indices for the estimation of group-specific $p \times p$ association matrices (and re-estimation of association matrices for pseudo-value calculations later).

```
# Note: Again, we will use a toy example with the first 30 out of 138 taxa.
OTUtab = combinedamgut[ , 8:37]

# Clinical/demographic covariates (phenotypic data):
# Note: All of these covariates in phenodat below will be included in the regression
#       when you use SOHPIE_DNA function later. Please make sure
```

```r
#       phenodat below include variables that will be analyzed only.
phenodat = combinedamgut[, 1:7] # first column is ID, so not using it.
```

```r
# Obtain indices of each grouping factor.
# In this example, a variable indicating the status of living with a dog was chosen (i.e. bin_dog).
# Accordingly, Groups A and B imply living without and with a dog, respectively.
newindex_grpA = which(combinedamgut$bin_dog == 0)
newindex_grpB = which(combinedamgut$bin_dog == 1)
```

Fit a pseudo-value regression via SOHPIE_DNA() function:

Upon our data processing step above is complete, we can then fit a pseudo-value regression using `SOHPIE_DNA` function. An important note! Please provide the object name of each OTU table and clinical/demographic data (i.e. metadata) separately in the function. In addition, you must indicate the object names of the indices for each group of a binary indicator variable that is used as a main predictor variable (e.g. living with a dog vs. without a dog). Lastly, you must enter a trimming proportion `c`, which ranges from 0.5 to 1.

```r
SOHPIEres <- SOHPIE_DNA(OTUdat = OTUtab, clindat = phenodat,
                        groupA = newindex_grpA, groupB = newindex_grpB, c = 0.5)
```

Additional features available in SOHPIE package:

Now, I would like to show you that SOHPIE has some convenient tools/functions after fitting a pseudo-value regression. There are functions that you can quickly extract names of taxa that are significantly differentially connected (DC; `DCtaxa_tab`), as well as p-values (`pval` and `pval_specific_var`), adjusted p-values (q-values; `qval` and `qval_specific_var`), coefficient estimates (`coeff` and `coeff_specific_var`), and standard errors (`stderrs` and `stderrs_specific_var`) of all variables that are considered in the regression or a specific variable.

```r
# qval() function will get you a table with q-values.
qval(SOHPIEres)
#>           bin_dog         age        sex bin_floss bin_exercise cat_alcohol1
#> 326792 0.33612263 0.903166021 0.10096355         1    0.8248660   0.24490111
#> 348374 0.24243370 0.001100184 0.16029320         1    0.7740866   0.64781516
#> 181016 0.33289325 0.887399324 0.20693215         1    0.8044906   0.81431616
#> 191687 0.38139833 0.887947631 0.44536421         1    0.8370341   0.22961569
#> 305760 0.30632901 0.880063017 0.11195699         1    0.8434169   0.64458179
#> 326977 0.28803043 0.793073185 0.11869984         1    0.8099179   0.75829937
#> 194648 0.10738956 0.892099575 0.51970935         1    0.7035875   0.50229826
#> 28186  0.25020128 0.226185628 0.12185274         1    0.8347916   0.70293919
#> 541301 0.42159900 0.834153248 0.01075535         1    0.7197917   0.08191323
#> 198941 0.01263389 0.853839486 0.10890326         1    0.7086346   0.08191323
#> 353985 0.02539901 0.866051916 0.24580990         1    0.7968984   0.75031008
#> 187524 0.33015864 0.411448070 0.28123507         1    0.7767193   0.60724327
#> 182054 0.37484369 0.537411116 0.48914893         1    0.4159067   0.72133725
#> 175537 0.09601170 0.805200995 0.41544659         1    0.4272540   0.31184746
#> 9753   0.31309380 0.882389067 0.10984350         1    0.3451530   0.70012519
#> 194211 0.04390943 0.841082160 0.40433708         1    0.4643529   0.82067831
#> 188518 0.10674886 0.853481324 0.12127550         1    0.7601830   0.36045249
#> 189396 0.38778526 0.802024465 0.12082849         1    0.4639709   0.69508384
#> 90487  0.26219312 0.768860824 0.08669701         1    0.6540847   0.58120117
#> 203708 0.43654526 0.890129605 0.15549082         1    0.7111461   0.64332479
#> 173965 0.32989140 0.893958131 0.37471540         1    0.8381836   0.57179105
#> 194661 0.32609146 0.872038423 0.10836615         1    0.8104509   0.80117550
#> 512309 0.20411613 0.880003663 0.45484395         1    0.7482363   0.81010387
```

```
#> 170124 0.34536058 0.836151713 0.46022485          1     0.4618909    0.79066209
#> 216862 0.34319112 0.681623038 0.07051945          1     0.4680569    0.08191323
#> 352304 0.17780567 0.492257005 0.04251456          1     0.5878969    0.31886273
#> 191306 0.27936406 0.211190039 0.42583971          1     0.4968951    0.36960141
#> 191541 0.33152662 0.913944007 0.11917020          1     0.4805421    0.41218991
#> 191547 0.32379054 0.915161953 0.48062051          1     0.7686641    0.78717487
#> 195493 0.34042111 0.847965205 0.01075535          1     0.3451530    0.69553944
#>        cat_alcohol2 bin_migraine
#> 326792    0.6920229   0.29537199
#> 348374    0.7813698   0.55161098
#> 181016    0.8231343   0.32441968
#> 191687    0.4913282   0.33207952
#> 305760    0.6795667   0.60162212
#> 326977    0.8391475   0.51171030
#> 194648    0.4646269   0.34390788
#> 28186     0.8308480   0.50722565
#> 541301    0.8489282   0.30224414
#> 198941    0.4994593   0.44435519
#> 353985    0.7829494   0.25469739
#> 187524    0.2744750   0.27116703
#> 182054    0.6290837   0.22052686
#> 175537    0.2744750   0.17024029
#> 9753      0.6702022   0.33760148
#> 194211    0.7369615   0.54653714
#> 188518    0.2744750   0.43481716
#> 189396    0.5057786   0.61093791
#> 90487     0.4046941   0.07355473
#> 203708    0.7454668   0.16668925
#> 173965    0.2744750   0.15259292
#> 194661    0.6681665   0.55332877
#> 512309    0.7184209   0.56174110
#> 170124    0.8193407   0.39953439
#> 216862    0.3885440   0.17079890
#> 352304    0.8336343   0.34523507
#> 191306    0.8193198   0.22129316
#> 191541    0.8149762   0.13321206
#> 191547    0.8423175   0.60178893
#> 195493    0.6910486   0.20377137
```

qval_specific_var function will be useful to retrieve the q-values of a specific variable, bin_dog in this example.

```
# Create an object to keep the table with q-values.
qvaltab <- qval(SOHPIEres)
# Retrieve a vector of q-values for a single variable of interest.
qval_specific_var(qvaltab = qvaltab, varname = "bin_dog")
#>           bin_dog
#> 326792 0.33612263
#> 348374 0.24243370
#> 181016 0.33289325
#> 191687 0.38139833
#> 305760 0.30632901
#> 326977 0.28803043
#> 194648 0.10738956
```

```
#> 28186  0.25020128
#> 541301 0.42159900
#> 198941 0.01263389
#> 353985 0.02539901
#> 187524 0.33015864
#> 182054 0.37484369
#> 175537 0.09601170
#> 9753   0.31309380
#> 194211 0.04390943
#> 188518 0.10674886
#> 189396 0.38778526
#> 90487  0.26219312
#> 203708 0.43654526
#> 173965 0.32989140
#> 194661 0.32609146
#> 512309 0.20411613
#> 170124 0.34536058
#> 216862 0.34319112
#> 352304 0.17780567
#> 191306 0.27936406
#> 191541 0.33152662
#> 191547 0.32379054
#> 195493 0.34042111
```

DCtaxa_tab will return a list containing of (1) names and q-values of taxa that are significantly DC between two biological conditions and (2) names of DC taxa only.

```
# Please do NOT forget to provide the name of variable in DCtaxa_tab(groupvar = )
# and the level of significance (0.3 in this example).
DCtaxa_tab <- DCtaxa_tab(qvaltab = qvaltab, groupvar = "bin_dog", alpha = 0.3)
DCtaxa_tab
#> $DCtaxa_complete_tab
#>           bin_dog
#> 348374 0.24243370
#> 326977 0.28803043
#> 194648 0.10738956
#> 28186  0.25020128
#> 198941 0.01263389
#> 353985 0.02539901
#> 175537 0.09601170
#> 194211 0.04390943
#> 188518 0.10674886
#> 90487  0.26219312
#> 512309 0.20411613
#> 352304 0.17780567
#> 191306 0.27936406
#>
#> $DCtaxa_names_only
#>  [1] "348374" "326977" "194648" "28186"  "198941" "353985" "175537" "194211"
#>  [9] "188518" "90487"  "512309" "352304" "191306"
```

Example II: Diet Exchange Study Data

```
data(combineddietswap)

OTUtab = combineddietswap[ , 5:ncol(combineddietswap)]
phenodat = combineddietswap[ , 1:4] # first column is ID, so not using it.
```

```
# Obtain indices for each groups
# (i.e. African-Americans from Pittsburgh (AAM) vs. Africans from rural South Africa (AFR))
# at baseline (time = 1) and at 29-days (time = 6)

# Group A1 for AAM at baseline.
newindex_A1 = which(combineddietswap$timepoint == 1 & combineddietswap$nationality == "AAM")
# Group A6 for AAM at 29-days.
newindex_A6 = which(combineddietswap$timepoint == 6 & combineddietswap$nationality == "AAM")
# Group B1 for AFR at baseline.
newindex_B1 = which(combineddietswap$timepoint == 1 & combineddietswap$nationality == "AFR")
# Group A6 for AFR at 29-days.
newindex_B6 = which(combineddietswap$timepoint == 6 & combineddietswap$nationality == "AFR")
```

We are done with loading the data and obtaining indices for each group for each time point. We then move onto the analysis step!

The first step is to estimate and re-estimate association matrices for each of groups A1, A6, B1, and A6 shown above. Of note, a list output comprises `data.frame` objects as `assomat` and `reest.assomat`.

```
est_asso_matA1 = asso_mat(OTUdat=OTUtab, group=newindex_A1)
est_asso_matA6 = asso_mat(OTUdat=OTUtab, group=newindex_A6)
est_asso_matB1 = asso_mat(OTUdat=OTUtab, group=newindex_B1)
est_asso_matB6 = asso_mat(OTUdat=OTUtab, group=newindex_B6)

## For each group, we take the difference of estimated
## association matrices between time points (29-days minus baseline).
asso_mat_diffA61 = est_asso_matA6$assomat - est_asso_matA1$assomat
asso_mat_diffB61 = est_asso_matB6$assomat - est_asso_matB1$assomat

## Similarly, for each group, we take the difference of re-estimated
## association matrices between time points (29-days minus baseline).
asso_mat_drop_diffA61 = mapply('-', est_asso_matA6$reest.assomat,
                               est_asso_matA1$reest.assomat, SIMPLIFY=FALSE)
asso_mat_drop_diffB61 = mapply('-', est_asso_matB6$reest.assomat,
                               est_asso_matB1$reest.assomat, SIMPLIFY=FALSE)
```

Then, for each group, we calculate changes in network centrality of each taxa between the association matrices estimated from the whole data and between the association matrices re-estimated from the leave-one-out sample.

```
## For changes in network centrality between association matrices estimated from the whole data.
thetahat_grpA = thetahats(asso_mat_diffA61)
thetahat_grpB = thetahats(asso_mat_diffB61)
## For changes in network centrality between association matrices
## re-estimated from the leave-one-out sample.
thetahat_drop_grpA = sapply(asso_mat_drop_diffA61, thetahats)
thetahat_drop_grpB = sapply(asso_mat_drop_diffB61, thetahats)
```

Next, we calculate jackknife pseudo-values.

```r
# Sample sizes for each group.
n_A <- length(newindex_A1)
n_B <- length(newindex_B1)

# Jackknife pseudo-values.
thetatilde_grpA = thetatildefun(thetahat_grpA, thetahat_drop_grpA, n_A)
thetatilde_grpB = thetatildefun(thetahat_grpB, thetahat_drop_grpB, n_B)

thetatilde = rbind(thetatilde_grpA, thetatilde_grpB)
```

Fit a robust regression regressing covariates on pseudo-values. Please note that the metadata/phenotype data should contain a set of predictors to be fitted only, so choose them wisely. Additionally, a trimming proportion `c` of the least trimmed squares estimator should be entered between 0.5 and 1.

```r
fitmod = pseudoreg(pseudoval=thetatilde, clindat=phenodat, c=0.5)
```

Lastly, we can extract summary results from the fitted model from `fitmod` object above. A list of `data.frame` objects for coefficient estimates, p-values, and q-values will be available.

```r
summary.result = pseudoreg.summary(pseudo.reg.res=fitmod,
                                   taxanames=colnames(OTUtab))

## In this study, the main grouping variable was nationality.
# We can use qval_specific_var to see the q-values only.
# of nationality. Alternatively, we further use DCtaxa_tab to see
# the DC taxa with their p-values.
qval_specific_var(summary.result$q_values, varname = "nationalityAFR")
#>                                      nationalityAFR
#> Akkermansia                              0.61145046
#> Alcaligenes faecalis et rel.             0.63453316
#> Allistipes et rel.                       0.34918397
#> Anaerostipes caccae et rel.              0.50827129
#> Anaerotruncus colihominis et rel.        0.29572525
#> Anaerovorax odorimutans et rel.          0.53637457
#> Aquabacterium                            0.63147406
#> Atopobium                                0.37148088
#> Bacillus                                 0.36151328
#> Bacteroides fragilis et rel.             0.52452524
#> Bacteroides intestinalis et rel.         0.47539357
#> Bacteroides ovatus et rel.               0.40801488
#> Bacteroides plebeius et rel.             0.13443267
#> Bacteroides splachnicus et rel.          0.27704830
#> Bacteroides stercoris et rel.            0.40009511
#> Bacteroides uniformis et rel.            0.28270019
#> Bacteroides vulgatus et rel.             0.50833482
#> Bifidobacterium                          0.32612327
#> Bilophila et rel.                        0.29483443
#> Brachyspira                              0.49468797
#> Bryantella formatexigens et rel.         0.34224595
#> Bulleidia moorei et rel.                 0.61642750
#> Burkholderia                             0.07491112
#> Butyrivibrio crossotus et rel.           0.30767022
#> Campylobacter                            0.29537417
#> Catenibacterium mitsuokai et rel.        0.17247097
#> Clostridium (sensu stricto)              0.58628729
```

```
#> Clostridium cellulosi et rel.              0.10078260
#> Clostridium colinum et rel.                0.25160069
#> Clostridium difficile et rel.              0.34701890
#> Clostridium leptum et rel.                 0.23450824
#> Clostridium nexile et rel.                 0.21446134
#> Clostridium orbiscindens et rel.           0.09975822
#> Clostridium ramosum et rel.                0.44440486
#> Clostridium sphenoides et rel.             0.45571811
#> Clostridium stercorarium et rel.           0.32897067
#> Clostridium symbiosum et rel.              0.41664021
#> Collinsella                                0.35770784
#> Coprobacillus catenaformis et rel.         0.12955950
#> Coprococcus eutactus et rel.               0.23067613
#> Corynebacterium                            0.50391899
#> Desulfovibrio et rel.                      0.49667775
#> Dialister                                  0.25425758
#> Dorea formicigenerans et rel.              0.14327946
#> Eggerthella lenta et rel.                  0.40838356
#> Enterobacter aerogenes et rel.             0.28481386
#> Enterococcus                               0.36741768
#> Escherichia coli et rel.                   0.26249783
#> Eubacterium biforme et rel.                0.28934508
#> Eubacterium cylindroides et rel.           0.47266968
#> Eubacterium hallii et rel.                 0.02502555
#> Eubacterium limosum et rel.                0.26174118
#> Eubacterium rectale et rel.                0.23542596
#> Eubacterium siraeum et rel.                0.25599774
#> Eubacterium ventriosum et rel.             0.03321701
#> Faecalibacterium prausnitzii et rel.       0.22065194
#> Fusobacteria                               0.08886839
#> Haemophilus                                0.62967744
#> Helicobacter                               0.41138701
#> Klebisiella pneumoniae et rel.             0.13453399
#> Lachnobacillus bovis et rel.               0.20620896
#> Lachnospira pectinoschiza et rel.          0.60681743
#> Lactobacillus catenaformis et rel.         0.61124668
#> Lactobacillus gasseri et rel.              0.08918605
#> Lactobacillus plantarum et rel.            0.62142059
#> Lactobacillus salivarius et rel.           0.52998399
#> Lactococcus                                0.29413476
#> Leminorella                                0.23164989
#> Megamonas hypermegale et rel.              0.61906807
#> Megasphaera elsdenii et rel.               0.36905014
#> Mitsuokella multiacida et rel.             0.36979380
#> Moraxellaceae                              0.26675811
#> Oceanospirillum                            0.08343851
#> Oscillospira guillermondii et rel.         0.29712894
#> Outgrouping clostridium cluster XIVa       0.12100754
#> Oxalobacter formigenes et rel.             0.02502555
#> Papillibacter cinnamivorans et rel.        0.20343172
#> Parabacteroides distasonis et rel.         0.53905646
#> Peptococcus niger et rel.                  0.59682391
#> Peptostreptococcus micros et rel.          0.56775886
```

```
#> Phascolarctobacterium faecium et rel.     0.63955084
#> Prevotella melaninogenica et rel.         0.60724637
#> Prevotella oralis et rel.                 0.62787186
#> Prevotella ruminicola et rel.             0.02502555
#> Prevotella tannerae et rel.               0.58507877
#> Propionibacterium                         0.26544624
#> Proteus et rel.                           0.27160399
#> Roseburia intestinalis et rel.            0.10006937
#> Ruminococcus bromii et rel.               0.59543212
#> Ruminococcus callidus et rel.             0.62196517
#> Ruminococcus gnavus et rel.               0.08945948
#> Ruminococcus lactaris et rel.             0.62404824
#> Ruminococcus obeum et rel.                0.18366518
#> Serratia                                  0.62348902
#> Sporobacter termitidis et rel.            0.62658135
#> Staphylococcus                            0.02502555
#> Streptococcus bovis et rel.               0.63946433
#> Streptococcus intermedius et rel.         0.11992018
#> Streptococcus mitis et rel.               0.08728281
#> Subdoligranulum variable at rel.          0.35819408
#> Sutterella wadsworthia et rel.            0.55710650
#> Tannerella et rel.                        0.43038520
#> Uncultured Bacteroidetes                  0.58630631
#> Uncultured Clostridiales I                0.63909382
#> Uncultured Clostridiales II               0.02502555
#> Uncultured Mollicutes                     0.32000238
#> Uncultured Selenomonadaceae               0.54286336
#> Veillonella                               0.53915668
#> Vibrio                                    0.42409690
#> Weissella et rel.                         0.23005923
#> Xanthomonadaceae                          0.37538957
#> Yersinia et rel.                          0.12776003
DCtaxa_tab(summary.result$q_values, groupvar = "nationalityAFR", alpha=0.05)
#> $DCtaxa_complete_tab
#>                                 nationalityAFR
#> Eubacterium hallii et rel.          0.02502555
#> Eubacterium ventriosum et rel.      0.03321701
#> Oxalobacter formigenes et rel.      0.02502555
#> Prevotella ruminicola et rel.       0.02502555
#> Staphylococcus                      0.02502555
#> Uncultured Clostridiales II         0.02502555
#>
#> $DCtaxa_names_only
#> [1] "Eubacterium hallii et rel."      "Eubacterium ventriosum et rel."
#> [3] "Oxalobacter formigenes et rel." "Prevotella ruminicola et rel."
#> [5] "Staphylococcus"                 "Uncultured Clostridiales II"
```

References

[1] Ahn S, Datta S. (2023). Differential Co-Abundance Network Analyses for Microbiome Data Adjusted for Clinical Covariates Using Jackknife Pseudo-Values. Under Review at *BMC Bioinformatics*.

[2] McDonald D. et al. (2018). American Gut: an Open Platform for Citizen Science Microbiome Research. *mSystems*. **3**(3), e00031–18

[3] O'Keefe SJ. et al. (2015). Fat, fibre and cancer risk in African Americans and rural Africans. *Nat Commun*. **6**, 6342
