# Supplementary material for: SOHPIE: statistical approach via pseudo-value information and estimation for differential network analysis of microbiome data
Source: Bioinformatics. 2023 Dec 22;40(1):btad766. doi: 10.1093/bioinformatics/btad766 (PMC10807904; doi:10.1093/bioinformatics/btad766)
Supplement: btad766_Supplementary_Data [file btad766_supplementary_data.zip › SupplementaryFile2_Sensitivity_Analysis.pdf]

Supplementary Table 1. For the sensitivity analysis of trimming proportion c for the robust regression, we have repeated the simulation study with 1000 replicates, described in our methodology paper. As an illustrative purpose, the SOHPIE was applied under the multivariable setting with the following specifications: group-specific effect sizes $\delta_1 = 0.05$ and $\delta_2 = 0.2$; network size $p = 20$; and sample size $n = 20$. The average operation time per simulation scenario was 33 minutes. The simulation experiment was performed on MacBook Pro 2019 with 2.4GHz Intel Core i5 CPU with 16GB RAM.

c	Precision	Recall	F1	Accuracy
0.5	0.51	0.69	0.57	0.51
0.6	0.51	0.70	0.57	0.51
0.7	0.51	0.69	0.57	0.51
0.8	0.51	0.70	0.57	0.51
0.9	0.51	0.68	0.56	0.51
1.0	0.52	0.62	0.54	0.51
